# Metastatic colonic and gastric polyps from breast cancer resembling hyperplastic polyps

**DOI:** 10.1186/s40792-018-0433-4

**Published:** 2018-03-23

**Authors:** Yoshiya Horimoto, Tetsuro Hirashima, Atsushi Arakawa, Hiroyoshi Miura, Mitsue Saito

**Affiliations:** 1 Department of Breast Oncology, Juntendo University School of Medicine, 2-1-1 Hongo, Bunkyo-ku, Tokyo, 113-0033 Japan; 2 Department of Pathology and Oncology, Juntendo University School of Medicine, 2-1-1 Hongo, Bunkyo-ku, Tokyo, 113-0033 Japan; 3 Tamaplaza South Gastrointestinal Clinic, 3-14-12 Shinishikawa, Aoba-ku, Yokohama-shi, Kanagawa 225-0003 Japan; 4 Department of Human Pathology, Juntendo University School of Medicine, 2-1-1 Hongo, Bunkyo-ku, Tokyo, 113-0033 Japan; 5 Department of Surgery, Koshigaya Municipal Hospital, 10-47-1 Higashikoshigaya, Koshigaya, Saitama 343-8577 Japan

**Keywords:** Breast cancer, Lobular carcinoma, Colonic metastasis, Mucosal metastatic polyp

## Abstract

Breast cancer metastasis to the gastrointestinal tract is relatively rare and is generally found when patients complain of symptoms such as gastrointestinal obstruction. Herein, we report a case with metastatic colonic and gastric lesions from breast cancer, with the formation of mucosal polyps which resembled typical hyperplastic polyps.

A 47-year-old woman underwent curable surgery for breast cancer and received standard systemic treatments. Her primary tumor was composed of a mix of invasive lobular and ductal carcinomas. During adjuvant endocrine therapy, she developed multiple colonic metastases, identified by colonoscopy performed as part of a general health check-up. She had no symptoms. Small elevated sessile polyps in the transverse colon and rectum showed histological features of signet-ring cell type adenocarcinoma, similar to the invasive lobular component of the primary breast cancer. During treatments for recurrent disease, she also developed multiple gastric metastases, with the same endoscopic and pathological features as the colonic lesions. Her treatment regimen was switched to oral chemotherapy, and she has since maintained stable disease for nearly 3 years. Multiple bone metastases eventually developed, and she was again switched to another systemic treatment but, to date, has remained free of symptoms.

We emphasize that the endoscopic findings of the metastatic lesions in the colon and stomach in this case highly resembled hyperplastic polyps. Since biopsy is not always performed for hyperplastic polyps in the gastrointestinal tract, we believe that this case report may encourage endoscopists to offer biopsies to the patient who has a history of breast cancer.

## Background

Breast cancer rarely metastasizes to the gastrointestinal (GI) tract, and only 5% of patients with recurrent disease have GI metastasis [1]. Invasive lobular carcinoma (ILC), which is characterized by minimal cell-cell adhesion, is known to more often metastasize to the GI tract than invasive ductal carcinoma (IDC), and this is especially true of ILC of the signet-ring cell type [2, 3].

In making the differential diagnosis of metastatic disease, several immunohistochemical (IHC) markers are useful for identifying breast cancer as the primary tumor. Estrogen receptor (ER) and human epidermal growth factor receptor 2 (HER2) are often examined, but their expressions are detected in 12–25% and 20% of gastric cancers [4–6], and in 30–56% and 4–14% of colon cancers [7–10], respectively. Thus, these examinations are not sufficient, in terms of tissue specificity, for identifying the origin of metastasis. CK7 is often observed in the epithelial layers of breast ducts and the lungs, while intestinal tissues show no expression of this protein [11, 12]. On the contrary, CK20 is often expressed in the large intestine and bile ducts, while it is rarely seen in the breast [11, 12]. Thus, metastatic disease from breast cancer is expected to be CK7-positive and CK20-negative, while the opposite is generally seen in primary colon cancer. GCDFP-15 and mammaglobin are also widely used. GCDFP-15, one of the proteins comprising the walls of benign cysts of the breast, is expressed in 32–47% of breast cancer metastatic lesions [13, 14]. Mammaglobin expression, observed only in breast and skin tissues, is also positive in 42–87% of metastases from breast cancer [13–15].

GI metastasis is typically found when patients complain of symptoms such as GI obstruction. At this stage, GI tract metastasis of ILC reportedly shows macroscopic findings of linitis plastic in some cases, rather than forming a large, localized, and elevated lesion [16–20].

Herein, we report a rare case with metastatic colonic mucosal polyps from breast cancer, which had an entirely hyperplastic appearance, and produced no symptoms. Metastasis localized only in the GI mucosa, verified endoscopically [18] or surgically [19], is very rare.

## Case presentation

**Fig. 1:**
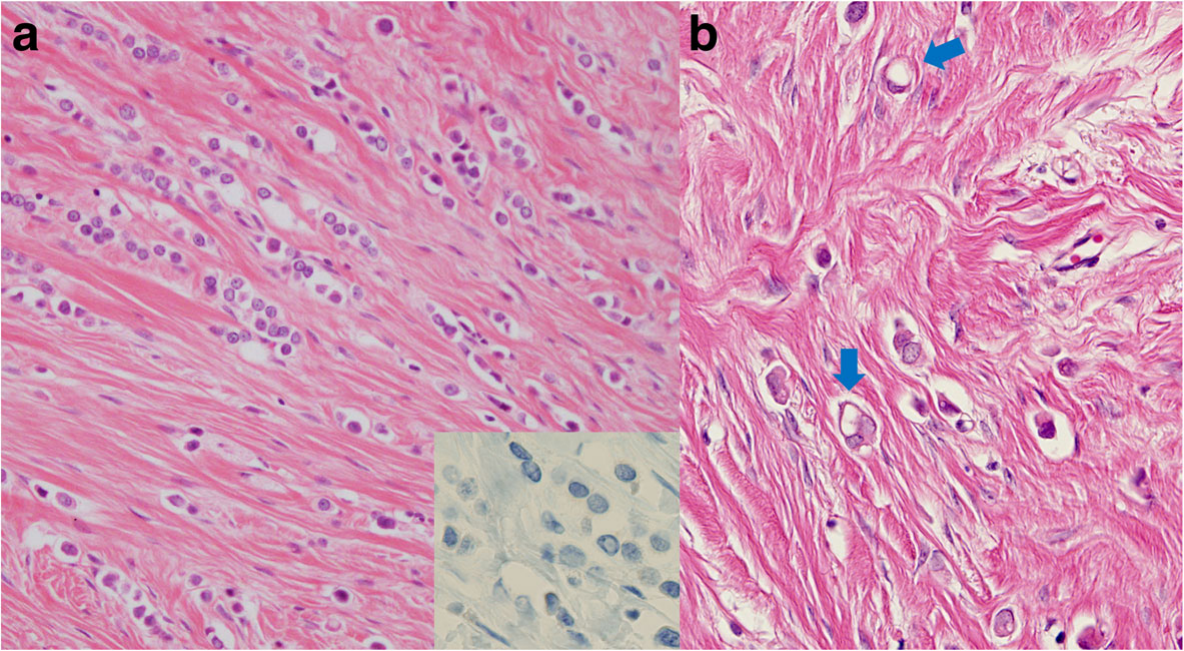
Pathological findings of primary breast cancer. **a** Invasive lobular carcinoma in primary breast lesion: discohesive small round cells (E-cadherin negative) are arranged in slender strands (Hematoxylin and eosin staining (HE), × 400). **b** Signet-ring cell features (blue arrows) were observed in the invasive lobular carcinoma lesion of the primary breast cancer (× 400)

**Fig. 2:**
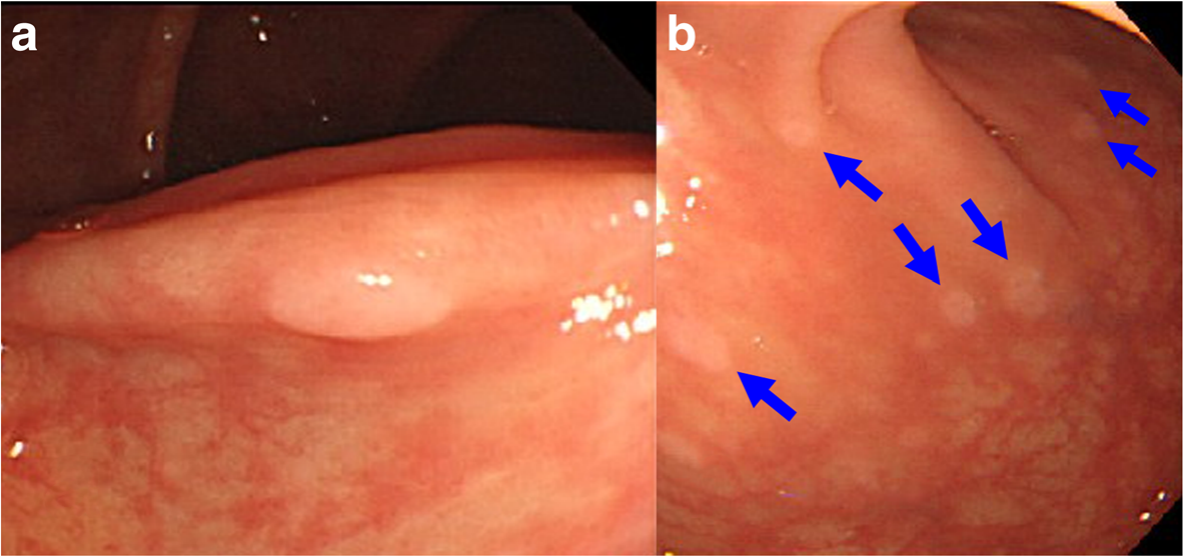
Metastatic mucosal polyp in transverse colon. **a** A small elevated sessile polyp (4 mm in diameter) was found in the transverse colon. Polypectomy was endoscopically performed and pathological examination revealed it to be a signet-ring cell carcinoma. **b** Multiple metastatic polyps in the transverse colon: re-examination by colonoscopy, performed 4 months after the diagnosis of metastasis, showed an increase in the numbers of metastatic polyps throughout the large intestine. Five biopsies were taken from the ascending colon, transverse colon, and rectum and all were confirmed to be signet-ring cell carcinoma

**Fig. 3:**
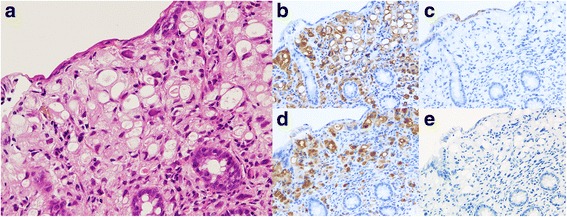
Pathological findings of metastatic colonic polyp (HE). **a** Adenocarcinoma (signet-ring cell type) in the mucosa of the transverse colon (× 200). **b**–**e** The metastatic mucosal polyp was CK7 positive, CK20 negative, GCDFP-15 positive, and mammaglobin negative (**b**, **c**, **d**, and **e**, respectively, × 200). These expressions corresponded to those of the primary breast cancer lesion

A 47-year-old woman felt a lump in her left breast and came to our hospital. We diagnosed invasive breast cancer (cT2N1M0 Stage IIB) and administered primary systemic chemotherapy (FEC × 4 cycles followed by Docetaxel × 4 cycles). Clinically, a complete response was obtained and she then underwent mastectomy (Bt+Ax). Pathological findings of the operative specimen were a mix of ILC and IDC. The ILC component had lost E-cadherin expression, and some cells showed signet-ring cell carcinoma features (Fig. 1a,b). Pathologically, the tumor was 105 mm in diameter and metastasis was detected in one lymph node; the histological features were consistent with ILC, while no lymphovascular involvement was detected (pT3N1aM0 Stage IIIA). The chemotherapy effect was grade 2, according to the general rules for Clinical and Pathological Recording of Breast Cancer (17th edition) by the Japanese Breast Cancer Society. ER and progesterone receptor (PR) were both positive and HER2 was negative. These IHC results were consistent with both the IDC and the ILC components. Following surgery, she received adjuvant endocrine therapy with a selective estrogen receptor modulator (SERM) for 2 years followed by sequential aromatase inhibitor (AI). Since she suffered from fatigue, the treatment was switched to SERM 4 years and 4 months after surgery. Eight months later, she underwent colonoscopy at another hospital as part of a general health check-up. She had no symptoms. A small elevated sessile polyp (4 mm in diameter) was found in the transverse colon (Fig. 2a) and was removed endoscopically. Histologically, adenocarcinoma (signet-ring cell type) was observed in the mucosa of the transverse colon (Fig. 3a). Because the histological type was not one commonly seen in colon cancer cases, biopsy specimens were reviewed at our hospital and compared with that of the primary breast cancer. Immunohistochemical examination revealed the polyp to be ER positive, PR negative, HER2 negative, E-cadherin negative, Cytokeratin (CK) 7 positive, CK20 negative, gross cystic disease fluid protein (GCDFP-15) positive, and mammaglobin negative (Fig. 3a–e). Based on histological similarity and the results of additional IHC examinations, we concluded that the polyp represented metastasis from the original breast cancer. We performed computed tomography and positron emission tomography at the time of diagnosis of recurrent disease, but no distant metastases, including the colonic polyp, were detected by these examinations.

**Fig. 4:**
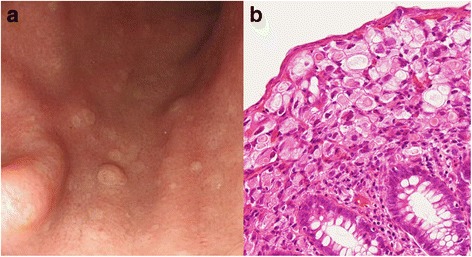
Multiple metastatic gastric polyps. Gastrointestinal fiberscopy revealed multiple small white elevated polyps in the stomach (**a**), which had not been seen at the time of the initial diagnosis of recurrent disease of the colon. Pathological findings (**b**, HE, × 200) confirmed signet-ring cell carcinoma

Since the metastases were diagnosed 8 months after starting SERM, which had been preceded by AI as adjuvant therapy, the patient restarted treatment with the initial AI. Colonoscopy, performed 2 months later, revealed that the numbers of small polyps had increased and were present throughout the large intestines (Fig. 2b). Five biopsies in total were taken from the ascending and transverse portions of the colon and the rectum, and all showed the same histological features as the signet-ring cell carcinoma polyp. However, on re-examination 4 months later, the numbers of polyps had obviously decreased, indicating the endocrine treatment to be effective. The patient maintained a stable disease state with this treatment for 1 year after the initial diagnosis of metastases. However, she eventually developed multiple small white elevated lesions (Fig. 4a), which had not been present at the time of initial diagnosis of the recurrent disease. We confirmed these gastric lesions to have the same pathological features, signet-ring cell type adenocarcinoma (Fig. 4b), as the colonic lesions, despite having the appearance of hyperplastic polyps. The gastric polyps showed the same expression pattern, with CK7, CK20, GCDFP-15, and mammaglobin being positive, negative, positive, and negative, respectively. She started treatment with capecitabine, and the gastric lesions remained stable for 2 years and 7 months, while the colonic lesions had almost disappeared within 10 months of starting this drug. She eventually developed multiple bone metastases, and her systemic treatment was switched to another chemotherapeutic regimen.

Throughout her clinical course, she has remained free of symptoms, suffering neither abdominal pain nor melena.

## Conclusions

We could not ascertain whether the metastases represented mucosal lesions if the patient had not undergone a routine health check-up. Otherwise, she might have progressed to a severe condition, such as GI perforation or obstructive ileus. Instead, this patient has been free of symptoms for more than 5 years with adequate systemic therapies against recurrent diseases.

We emphasize that, based on the endoscopic findings, these colonic and gastric metastatic lesions essentially had the appearance of hyperplastic polyps. Since biopsy is not consistently performed for hyperplastic polyps in the GI tract, this case report may encourage endoscopists to offer biopsies to patients with a history of breast cancer. Whether or not this endoscopic appearance indicates early GI metastasis needs to be determined based on further case reports.

## Abbreviations

AI: Aromatase inhibitor
CK: Cytokeratin
ER: Estrogen receptor
GI: Gastrointestinal
HER2: Human epidermal growth factor receptor 2
IDC: Invasive ductal carcinoma
IHC: Immunohistochmical
ILC: Invasive lobular carcinoma
PR: Progesterone receptor
SERM: Selective estrogen receptor modulator
